# Protocol for SAMS (Support and Advice for Medication Study): A randomised controlled trial of an intervention to support patients with type 2 diabetes with adherence to medication

**DOI:** 10.1186/1471-2296-9-20

**Published:** 2008-04-11

**Authors:** Andrew J Farmer, A Toby Prevost, Wendy Hardeman, Anthea Craven, Stephen Sutton, Simon J Griffin, Ann-Louise Kinmonth, The Support and Advice for Medication Trial Group

**Affiliations:** 1 Department of Primary Health Care, University of Oxford, Oxford, OX3 7LF, UK; 2 General Practice and Primary Care Research Unit, Institute of Public Health, University of Cambridge, Cambridge, CB2 0SR, UK; 3 MRC Epidemiology Unit, Institute of Metabolic Science, Addenbrooke's Hospital, Cambridge, CB2 0QQ, UK

## Abstract

**Background:**

Although some interventions have been shown to improve adherence to medication for diabetes, results are not consistent. We have developed a theory-based intervention which we will evaluate in a well characterised population to test efficacy and guide future intervention development and trial design.

**Methods and Design:**

The SAMS (Supported Adherence to Medication Study) trial is a primary care based multi-centre randomised controlled trial among 200 patients with type 2 diabetes and an HbA1c of 7.5% or above. It is designed to evaluate the efficacy of a two-component motivational intervention based on the Theory of Planned Behaviour and volitional action planning to support medication adherence compared with standard care. The intervention is delivered by practice nurses. Nurses were trained using a workshop approach with role play and supervised using assessment of tape-recorded consultations. The trial has a two parallel groups design with an unbalanced three-to-two individual randomisation eight weeks after recruitment with twelve week follow-up. The primary outcome is medication adherence measured using an electronic medication monitor over 12 weeks and expressed as the difference between intervention and control in mean percentage of days on which the correct number of medication doses is taken. Subgroup analyses will explore impact of number of medications taken, age, HbA1c, and self-reported adherence at baseline on outcomes. The study also measures the effect of dispensing medication to trial participants packaged in the electronic medication-monitoring device compared with conventional medication packaging. This will be achieved through one-to-one randomisation at recruitment to these conditions with assessment of the difference between groups in self-report of medication adherence and change in mean HbA1c from baseline to eight weeks. Anonymised demographic data are collected on non-respondents. Central randomisation is carried out independently of trial co-ordination and practices using minimisation to adjust for selected confounders.

**Discussion:**

The SAMS intervention and trial design address weaknesses of previous research by recruitment from a well-characterised population, definition of a feasible theory based intervention to support medication taking and careful measurement to estimate and interpret efficacy. The results will inform practice and the design of a cost-effectiveness trial [ISRCTN30522359].

## Background

Diabetes is a major public health problem. The number of people with diabetes is estimated to reach 330 million by 2030 [1]. There is a high clinical and economic burden from the disease [2]: people with diabetes have a two-to-four fold increased risk of cardiovascular disease compared to the general population and increased incidence of retinopathy, peripheral nerve damage and renal problems.

Evidence supports the use of multiple medications to control blood glucose and cardiovascular risk among patients with type 2 diabetes [3], and, this may lead to prescriptions of eight or more medications a day. Up to half of this medication may not be taken as prescribed [4,5], with adherence to medication falling as dosage frequency rises [6]. Failure to take medication has important consequences. It not only reduces efficacy of the treatment, but wastes healthcare resources in prescribed pills not taken, extra consultations, referrals, investigations and hospital admissions [7,8]. The availability of an effective intervention to support patients with type 2 diabetes in taking their medication regularly would make a major contribution to human health.

A variety of interventions to support adherence to medication have been tested for efficacy [9], and their clinical application assessed [10]. Although some interventions are effective in improving adherence, the results are inconsistent. These studies have used complex packages of care, targeting multiple self-care activities and therefore limiting identification of factors that lead to and might modify non-adherence. Approaches to supporting behaviour change are now informed by evidence about the psychological determinants of behaviour, and techniques to alter them, and there is potential to apply these techniques in the field of medication adherence [11,12].

Adherence to medication can be defined in many ways. Our definition is the extent to which medicines are taken regularly as prescribed. We argue that to understand the determinants of adherence better and optimise the impact of different intervention components upon them, we need to design interventions based on clear conceptual frameworks. We also need to test whether targeting determinants of medication adherence (e.g., patient's beliefs) results in greater levels of adherence [11,13].

People may not take their medication because of ambivalence about pros and cons of adherence (intentional lapse). Others, who intend to take their medications regularly, may still forget to do so (non-intentional lapse). Intentional and non-intentional lapses in adherence have been identified as two important elements contributing to overall non-adherence [14]. Drawing on psychological theory and evidence [15,16], we have developed approaches to address these two elements: to increase patients' motivation to take their tablets regularly by targeting underlying beliefs; and to help patients define specific action plans to facilitate the translation of intentions into action and habit formation. The two components can be defined as motivational – targeting the cognitive determinants of intention – and volitional – defining action plans to implement the target behaviour [15].

The motivational component of the intervention is based on the Theory of Planned Behaviour (TPB) [16]. This theory specifies beliefs which may be targeted to strengthen intention. In particular techniques include reinforcement of positive beliefs and problem solving approaches to negative beliefs. The theory's constructs account for 35 to 50% of variance in intention and 26 to 35% of variance in behaviour [17]. This illustrates a gap between intention and behaviour. The use of action planning, also called implementation intentions[15], is a promising approach to bridge this gap. A range of studies has demonstrated that explicit consideration of the circumstances under which a behaviour will be enacted (action plans), promotes clinically important change in specific health related behaviours including consumption of vitamin C tablets, attendance for cervical cytology screening and breast self-examination [18].

In clinical trials evaluating medication taking, adherence in the control group is often high. This may be because patients willing to participate in trials are likely to be adherent, because self-report measures may overestimate true adherence, or because more objective measurement focuses attention, on this target behaviour [7,10]. To interpret the effects of interventions better, future trials need to characterise the population from which participants have been recruited by collecting data about non-responders, encouraging respondents who do not wish to take part in the trial to complete baseline questionnaires, and investigating the effect of measurement itself on adherence and clinical risk.

In previous pilot work we have developed measures and interventions [19] using the approach defined by the Medical Research Council Framework for development of interventions for evaluation in randomised trials [20,21]. We therefore intend to estimate the efficacy of support for medication adherence on medication taking behaviour using motivational and action planning techniques; and estimate the effect of monitoring medication-taking itself on self-reported adherence and glycaemic control in a randomised trial. The trial addresses important limitations in the literature by recruitment from a well-characterised population, definition of a feasible intervention to support medication taking, and careful measurement to estimate and interpret efficacy.

## Methods/Design

### Trial design

**Figure 1:**
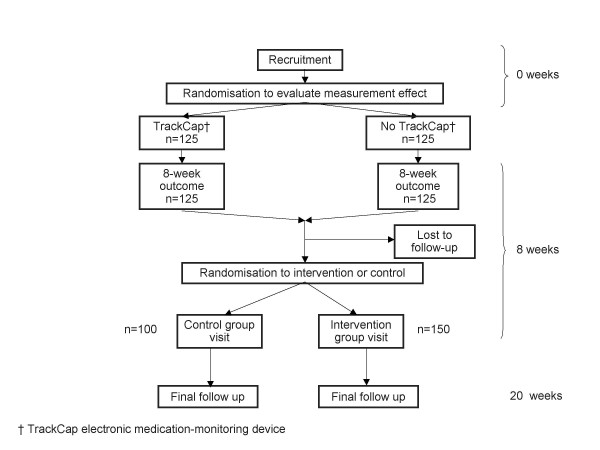
Flow of participants through study.

This parallel group trial has two sequential randomisations. The main randomisation takes place eight weeks after recruitment, is unbalanced and compares a two-component intervention addressing motivation and action planning with a control intervention in which patients only take part in data collection (Figure 1). Unbalanced randomisation is used to maximise experience with the intervention across a range of patients with follow up at twenty weeks. The measurement effect randomisation, which takes place at the baseline recruitment visit, is balanced and assesses the effect of dispensing medication in a container that records opening compared with dispensing medication in standard packaging: with follow up at eight weeks.

### Ethical approval

The London, multi-centre research ethics committee has reviewed and approved the protocol (06/MRE02/3).

### Setting

Recruitment of patients is from general practices in Oxfordshire, Milton Keynes, Suffolk, Essex and Huntingdonshire.

### Patients

Patients are eligible for inclusion in the trial if diagnosed aged 18 years or above with type 2 diabetes of at least three months duration, able to give informed consent, currently taking any oral glucose-lowering agent and with a HbA1c ≥ 7.5%. Those approached are deemed by their general practitioner able to complete a consent form, independent in medication taking, and appropriate for tight glycaemic control.

### Intervention

After an eight week period of initial observation, patients are allocated to either an intervention visit to support medication adherence, or to a control visit at which their current medication regimen is recorded.

The intervention was developed and piloted after a detailed study to identify beliefs held about diabetes [19]. The intervention visit is delivered by the practice nurse. In the first, motivational component of the intervention, the nurse elicits the patient's beliefs relevant to their intention to take medication regularly as prescribed based on the TPB. Nurses use scripts to guide the order and phrasing of questions that elicit individual beliefs about benefits and harms, views of important others and factors that may facilitate or inhibit taking medication regularly as prescribed. Positive beliefs are reinforced with provision of tailored information and problem solving is applied in relation to negative beliefs [16]. In the second, action planning component, the nurse asks the patient to write down the exact circumstances under which they will take their medication (using an "if.then" formulation to elicit where, when and how this will occur) [15]. In the control visit, delivered by the practice nurses, none of the above techniques are applied.

### Intervention fidelity

Initial training is provided over one day for the practice nurses delivering the intervention, using a workshop approach with role-play led by a psychologist and intervention facilitators involved in quality assurance of intervention delivery. It includes explanation of the rationale for the intervention, use of a detailed manual providing information about the theoretical base and operationalisation of the different components, how to use the intervention to establish agreement with the prescription plan, and use of a 'script' to promote fidelity of intervention delivery. Assessment of audiotapes of intervention sessions is conducted to assess fidelity of delivery using standardised forms. Subsequently, coaching and feedback is provided to the nurses to optimise intervention delivery. Possible sources of bias in intervention delivery are addressed with the practice nurses throughout training and ongoing support. These include the need to avoid (i) delivering the intervention to control group patients, (ii) discussing the intervention techniques with other members of the primary care team and (iii) using intervention techniques not specified in the script.

### Measurement effect

At the baseline visit patients are randomised to be dispensed their metformin tablets in a container with a lid that records the occurrence and timing of opening (TrackCap, Aardex, Zurich, Switzerland), or to continue taking their medication in standard packaging as before the trial. If participants are not taking metformin, another of their usual oral hypoglycaemic medication is dispensed in the electronic medication-monitoring device.

### Randomisation

Randomisation of patients to assess intervention and baseline measurement effects is carried out independently of trial co-ordination and intervention by the trial statistician. A partial minimisation procedure is used to dynamically adjust randomisation probabilities to balance the baseline stratification variables. For measurement effect, randomisation at baseline includes practice, duration of diabetes, HbA1c result from the practice record and patient baseline measure of self-report adherence. For intervention, randomisation at eight weeks includes the baseline measurement-effect randomisation group allocation, and baseline HbA1c is included instead of the practice record. Staff receiving and downloading the data from the electronic medication-monitoring device are blind to allocation, as are laboratory staff measuring HbA1c.

### Study procedures

#### Baseline data collection and randomisation to electronic medication taking measurement

Eligible patients registered with the practice are identified by the practice nurse. Baseline data from the notes is recorded by the practice nurse and anonymised to provide data from which to characterise the population from which participants were recruited. Eligible patients are sent a letter from the practice including study details, a questionnaire asking about basic demographics, medication regimen, self-reported medication adherence, and beliefs about taking diabetes medicines without missing a day (Table 1 and Table 2). If willing to help further, a phone call is made by the practice nurse to the patient to arrange a study appointment.

**Table 1:** Trial measures and timing

	Baseline visit	Before 8 week visit	At 8 week visit	After 8 week visit	Final visit at 20-weeks
Basic demographics	x				

Medication	x		x		x
Co-morbidity	x				
**Clinical measures**					
Weight	x				x
Blood pressure	x				x
HbA1c (Central laboratory)	x		x		x
**Adherence**					
Electronic medication monitor for preceding period			x (50%)		x
Self-report (MARS) Diabetes		x			x
Drug concentrations			x		x
**Psychological**					
TPB† Direct measures and intention	x	x		x	
TPB† Indirect measures	x	x		x	
Time and location of taking medicines					x
Communication with practice nurse					x
**Quality of Life**					
Short-Form 12‡	x				x
Diabetes Treatment Satisfaction Questionnaire					x
Hypoglycaemia			x		x

^‡^ 12-Item short form Medical Outcomes Study health survey questionnaire.

^†^ Theory of Planned Behaviour.

**Table 2:** Theory of Planned Behaviour measures of belief and intention towards taking oral glucose-lowering medications

**Variable name**	**Number of items†**	**Sample question**
Direct attitude	2	It is beneficial for me to take my diabetes medicines without missing a day
Direct subjective norm (injunctive)	2	Most people who are important to me think I should take my diabetes medicines without missing a day
Direct subjective norm (descriptive)	1	If they were taking part in this study, most people who are important to me would take their diabetes medicines without missing a day
Direct perceived behavioural control	3	It is difficult for me to take my diabetes medicines without missing a day
Indirect attitude	7	If I were to take my diabetes medicines without missing a day, it would keep my diabetes under control
Indirect subjective norm (injunctive)	3	Members of my family or close relatives would approve of me taking my diabetes medicines without missing a day
Indirect perceived behavioural control	4	Changes to my daily routine would make it more difficult for me to take my diabetes medicines without missing a day
Intention	2	I intend to take my diabetes medicines without missing a day

†Each measured on a 7-point scale from 1 = strongly disagree to 7 = strongly agree

Willing patients attend a baseline recruitment consultation of 40 minutes with their practice nurse. In advance of the visit patients are allocated to be dispensed medication in the electronic medication-monitoring device or in standard packaging. Informed consent is obtained and additional clinical data collected. Blood tests are taken, and questionnaires completed. For those allocated to the electronic medication-monitoring device, its use is explained, and the practice dispenser or pharmacist dispenses the patient's usual prescription for metformin or alternative oral glucose lowering agent in the device. For those allocated to standard packaging, the practice dispenser or pharmacist dispenses medication in standard blister-packs. A follow-up visit is arranged in eight weeks and, in advance of the visit, the patient is randomised to be allocated to receive the intervention or control visit.

#### Intervention and control visit

Prior to the eight-week study visit patients are sent a questionnaire to be completed at home and returned to the coordinating centre in a sealed envelope by post. At the eight-week visit, patients allocated to the intervention take part in a 50 minutes interview with the practice nurse including the intervention and data collection. The control visit lasts approximately 20 minutes in which the data are collected. All patients at the eight week visit have blood samples taken and inquiry made about any possible adverse events including hypoglycaemia. A postal questionnaire is completed one week after the eight week visit.

#### Follow up to 20 weeks

Final follow up for all patients at 20 weeks involves a visit to the practice nurse and includes a blood sample for measurement of HbA1c and drug concentrations, and a final questionnaire.

### Measurement

#### Primary outcome for intervention trial

The primary outcome for the intervention is the percentage of days on which the prescribed dose of main hypoglycaemic medication is taken as prescribed, measured with the TrackCap electronic medication monitor over a 12-week period from eight to twenty weeks.

#### Primary outcome for measurement-effect trial

The primary behavioural outcome for the measurement effect of the use of an electronic medication-monitoring device compared with standard packaging is change in self reported adherence measured with the Medication Adherence Report Schedule (MARS). The primary clinical outcome for the measurement effect is mean HbA1c at eight weeks.

#### Secondary outcomes for intervention trial

Secondary outcomes are HbA1c, well-being measured with the 12-item Short Form Medical Outcomes Study health survey questionnaire (Short Form-12) [22] and treatment satisfaction measured with the Diabetes Treatment Satisfaction Questionnaire (DTSQ) [23].

#### Additional measures of medication adherence

Other measures include serum drug concentrations and self-reported measures of medication adherence (MARS) [24], and dispensing records to complement electronic medication-monitoring. Drug concentrations will be collected to explore whether the electronic medication-monitoring device records of timing of doses relate to the presence of therapeutic concentrations at study visits.

#### Psychological and process measures

A questionnaire based on the Theory of Planned Behaviour is used to assess attitude, subjective norm, perceived behavioural control, and intention to take diabetes medicines without missing a day. The questionnaire also assesses perceived consequences of taking diabetes medication (behavioural beliefs); perceived views of significant others about taking diabetes medication (normative beliefs); perceived factors that make taking diabetes medication easier or difficult (control beliefs). These measures are based on our earlier pilot work [19].

We are using a measure to assess patients' ratings of communication with nurses (covering the ability to tell the nurse personal or troubling things and feeling understood) [25], and ask open-ended questions about when and where medications are usually taken. Tape recordings of consultations are used to assess fidelity of intervention delivery, using a-priori criteria. The intervention facilitators record adherence to techniques specified in the intervention script on standardised forms.

#### Baseline measures to characterise participants

Socio-demographic and clinical measures include measures of duration of diabetes, overall drug regimen and numbers of prescribed medication doses. For the purposes of characterising the eligible population and randomisation at baseline, the last practice-recorded HbA1c is also recorded.

### Sample size

The trial is planned to follow-up 200 patients, providing 80% power at the 5% significance level to detect a difference in means between randomised groups of 5% (1.5 days per month difference) in the percentage of days on which the correct number of doses is taken. This was based on an estimate of the standard deviation of this measure of 13.5% in a pilot study for the trial conducted in 2001 in Newmarket, Cambridgeshire (personal communication Dr Simon Griffin). Assuming a follow-up rate of 85%, a maximum of 250 participants would need to be recruited.

For the measurement effect we can detect a difference of one point in the MARS self-reported adherence measure (a small to moderate effect size) [24], with 80% power and a two sided test at the 5% level with a conservative estimate of a 2.5% standard deviation. We will also detect a 0.5% difference in HbA1c at eight weeks with 80% power at the 5% level assuming a standard deviation of 1.25% for HbA1c.

### Analysis

#### Intervention effect

Analysis will be by intention-to-treat using multiple imputation for those with missing data by the method of Rubin [26]. This will be supported by a sensitivity analysis reporting all cases where the primary outcome is available and using optimistic and pessimistic scenarios with the imputed estimates for missing data [27]. The mean percentage of days on which the correct number of doses is taken will be compared between the groups allocated to intervention and control, using a non-parametric bootstrap method to derive the difference in means with a 95% confidence interval [28,29].

#### Measurement effect

The primary behavioural outcome for the assessment of measurement effect is self-reported adherence (MARS) at eight weeks compared between groups allocated to use of the medication monitor compared to those allocated to dispensing of medicines in standard packaging. The primary clinical outcome is HbA1c at 8-weeks compared between groups. In order to increase the precision of estimated intervention effects continuous measures will be analysed with adjustment for the baseline covariate prior to the first randomisation, if available, with missing baseline data included by the missing indicator method [27].

#### Sub-group analyses

Subgroup analyses will be carried out to explore the impact of number of medications, age, HbA1c, self-reported adherence at baseline and prior randomisation to the electronic medication-monitoring device on the intervention effect.

#### Additional analyses

We will assess the impact of the intervention on beliefs about taking diabetes medication, and test whether any observed change in medication adherence is mediated by the intervention's impact on beliefs. We will explore the potential role of nurse communication style as a moderator of effect. We will compare the differences between participating and non-participating patients.

## Discussion

This trial is designed to estimate the efficacy of a two-component intervention targeting determinants of intentions and action planning as possible mediators of improved medication adherence. Measures and interventions have been developed using the approach defined by the Medical Research Council Framework for development of interventions for evaluation in randomised trials [20,21]. In addition to estimating efficacy the trial will provide information on psychological mechanisms of increasing medication adherence and assess the impact of trial measurement on outcomes.

We are conducting this trial in a well characterised primary care population. The training of nurses to deliver the intervention uses scripts and feedback from taped intervention sessions to maximise delivery as planned and consistent delivery across nurses and over time. We are able to test impact of the communication style of the practice nurse by examining the relationship between patient attitudes about communication and behavioural outcomes. We have optimised clear communication of the intervention through training and feedback to the nurses delivering the intervention.

This trial has been designed to address the weaknesses of previous work in the area of medication adherence. It approaches medication taking as a discrete behaviour and uses psychological theory and evidence in the design of intervention to address intentions and the step from intention to actions, and in measurement of the process. It will therefore be possible to explore the extent to which interventions that address these components of medication adherence might improve outcomes. Additional analyses will estimate the extent to which any observed effects of the intervention on behaviour are mediated by intention and action planning. The trial will explore the extent to which previous trials may have failed to show efficacy as a result of the kind of people who participate in such trials, and the kinds of measures of adherence used, including the impact of measurement itself on adherence and HbA1c.

This trial will provide one of the first evaluations of an intervention developed using psychological theory to support patients in adherence to diabetes medication. If the trial provides evidence of efficacy, it will add to the clinical approaches for medicines education currently in use.

If the two-component intervention shows evidence of efficacy, we propose to compare it with the action-planning only intervention in a future trial. Future research in this area will also need to incorporate detailed health economic assessments to assess cost-effectiveness. However, because of the large numbers of people with diabetes, even very small effects of an intervention on adherence offer potential for major public health gains.

## Competing interests

The author(s) declare that they have no competing interests.

## Authors' contributions

A-LK and AF led the grant-writing group. All authors were involved in the development and application of the protocol. The contributions of other members of the SAMS study team are gratefully acknowledged and listed above. AF is the guarantor of this paper.

## Funding

This trial is supported by the Medical Research Council and through National Health Service R&D support funding.

### The SAMS Team

*Writing group: *A Farmer, AT Prevost, W Hardeman, A Craven, S Sutton, S Griffin, A-L Kinmonth. *Coordinating Centre: *A Craven, J Oke, D White. *Trial Statistician: *AT Prevost. *Intervention Development: *A Farmer, W Hardeman, I Kellar, Y Kim, M Selwood, S Sutton. *Measures*: A Farmer, S Griffin, D Hughes, I Kellar, S Sutton. *Practices: *Suffolk; The Rookery Medical Centre Newmarket, Woolpit Health Centre; Huntingdonshire; Rainbow Surgery Ramsey, Ramsey Health Centre, The Surgery Papworth Everard, Spinney Surgery St. Ives, Eaton Socon Health Centre; Essex; John Tasker House Surgery Great Dunmow; Oxfordshire; Woodcote Surgery, Horse Fair Surgery Banbury, Islip Medical Practice; Milton Keynes; Parkside Medical Centre, Stantonbury Health Centre. *Pharmacies: *Lloyds Pharmacy Ramsey, Cox & Robinson Banbury; Yogi Pharmacy Great Dunmow; P & I Smith Bletchley, Cox & Robinson Bletchley, Lloyds Pharmacy Eaton Socon, McLaren Pharmacy New Bradwell. *Nurse training and intervention co-ordination: *S Boase, Y Kim. *Fidelity assessment: *J Argles, S Boase, P Gash, Y Kim, M Selwood. *Primary care network liaison*: J Graffy. *Pharmaceutical adviser: *S. Ashwell.

## Pre-publication history

The pre-publication history for this paper can be accessed here:


